# Graphitic Carbon Nitride as Reinforcement of Photopolymer Resin for 3D Printing

**DOI:** 10.3390/polym16030370

**Published:** 2024-01-29

**Authors:** Jong Wan Ko

**Affiliations:** 3D Printing Manufacturing Process Center, Smart Forming Process Group, Korea Institute of Industrial Technology (KITECH), Ulsan 44776, Republic of Korea; jwko@kitech.re.kr

**Keywords:** polymer additive manufacturing, digital light processing 3D printing, graphitic carbon nitride, reinforcement, dispersion process

## Abstract

Digital light processing (DLP) has the advantages of higher printing speed and product precision than other 3D printing technologies. However, DLP products have low mechanical strength owing to the inherent properties of photocurable materials. Graphitic carbon nitride (GCN), which is an abundant hydrogen bonding motif (-NH_2_, -NH), has low solubility in most solvents; thus, to use GCN as a reinforcement of the polymer matrix, optimal dispersion processes must be applied. In this study, GCN was proposed as a novel reinforcing material to improve the mechanical properties of photocurable epoxy acrylate (EA) resins for DLP. Herein, two-step (planetary mixing and ultrasonication) processes were applied to disperse GCN within EA, and the dispersion performance was identified by checking the degree of precipitation over time. To test the printability of the dispersed GCN/EA composites subjected to DLP 3D printing, cube specimens of GCN/EA composites were prepared, and the dispersed GCN/EA output had a low dimensional error of 0.3–1.3%, while the undispersed composite output showed larger dimensional errors of 27.7–36.2%. Additionally, in the mechanical test of the DLP-3D-printed sample (dispersed GCN/EA composite), the tensile strength and elastic modulus of the dispersed GCN/EA composite specimen were measured to be 75.56 MPa and 3396 MPa, respectively, which were improved by 22% (tensile strength) and 34% (modulus of elasticity) in relation to those of the neat EA specimen. This study is the first to use GCN as a reinforcement and manufacture a composite product for DLP with excellent performance (22% increased tensile strength) through the optimal dispersion of GCN. Considering the high mechanical performance, DLP products using the GCN/EA composites can be used in industries such as automobiles, shipbuilding, and aviation.

## 1. Introduction

Additive manufacturing (AM), which builds products layer-by-layer based on a computer-aided design (CAD) model, has gained significant attention in recent years. This process has remarkable advantages, such as production time and material reduction, and high design freedom over conventional manufacturing methods such as machining, injection molding, and casting [1,2,3,4]. 3D printing technologies have been studied for their application in the development of a wide range of materials, such as metals [5,6,7], ceramics [8,9,10], and polymers [11,12,13]. Shuai et al. printed a complex biocompatible scaffold that mimics the native rabbit trachea structure by using waterborne biodegradable polyurethanes through fused deposition modeling (FDM) 3D printing; their scaffold achieved a compression stress of 0.3–0.8 MPa, which is comparable with that of the native rabbit trachea [14].

Polymer 3D printing is the most attractive and emerging technology that allows the printing of low-cost, functional parts with adaptable properties [15]. 3D printing of polymer materials is typically classified into FDM, selective laser sintering (SLS), and vat photopolymerization. FDM is a common 3D printing method that uses an extrusion nozzle with a solid filament material, and SLS is a powder-based 3D printing method that uses a laser to melt the material. Vat photopolymerization has great advantages for higher resolution and smoother surfaces compared to FDM and SLS 3D printing methods [16,17]. Vat photopolymerization 3D printing, divided into stereolithography (SLA) and digital light processing (DLP), is an AM method that prints using the curing process of a photopolymer liquid resin. SLA 3D printing technology has a high resolution because of its usage of a laser scanning system to solidify the photopolymer resin with a relatively lower printing speed [18]. DLP 3D printing is the most widely used vat photopolymerization 3D printing due to its high precision and printing speed, applicability of printing area, and exceptionally smooth surface of printouts [19].

DLP 3D printing equipped with a digital micro-mirror device (DMD) chip that involves UV light irradiation of a plane unit of an entire layer and uses a photosensitive resin is a simpler, faster, and economical method than SLA 3D printing, which uses a laser as the light source [20,21]. Despite the fact that DLP 3D printing has considerable potential for mass production and is an ideal alternative to conventional fabrication processes [22], there are requirements that need to be met to materialize DLP 3D printing as a general fabrication method. DLP-3D-printed products have lower mechanical properties, especially tensile and flexural strength, than those manufactured by conventional fabrication methods such as compression molding and injection molding due to the limitation of crosslinking in photopolymer [23,24]. Recently, to enhance the mechanical properties of DLP-3D-printed products, several strategies based on process modification [25], composition modification [26], and the use of reinforcements [27] have been proposed.

Reinforcements commonly applied to improve the mechanical properties of DLP-3D-printed products include cellulose nanocrystals (CNCs) [28], carbon nanotubes [29], and graphene [30]. Li et al. used CNCs as a reinforcement material for a photopolymer matrix (polyethylene glycol diacrylate) and reported that the tensile strength increased by up to 50% in the composite with 1.0 wt% CNC content in relation to that of a pristine photopolymer specimen. In addition, Joo et al. added graphene sheets to a polyurethane resin for DLP 3D printing and confirmed that the tensile strength of the composite increased by 119% at 2 wt% (69.3 MPa) in relation to the pristine polyurethane output (31.6 MPa) [30]. However, to date, most of these reinforcements are non-economical, difficult to synthesize, and toxic, limiting their practical use in industries [31,32,33].

Graphitic carbon nitride (GCN), generally known as g-C_3_N_4_, is a polymeric material bearing interconnected tri-s-triazine units. Due to the inherent electrical characteristics of the medium band gap (2.7 eV) that responds to visible light, GCN has been particularly applied in the fields of photocatalysis [34,35,36,37,38,39], energy production [40,41,42,43,44], and photosensing [45,46]. Furthermore, g-C_3_N_4_ has several advantages, such as low cost, facile synthesis from nitrogen-rich precursors, eco-friendliness, and excellent chemical resistance. Another distinguishing property of GCN is the presence of numerous hydrogen bonding motifs (-NH_2_, -NH functional groups). Recently, several studies have reported that the mechanical properties of a composite were improved by adding GCN as a reinforcement in a specific matrix material, and interactions between GCN and the matrix strengthened owing to the formation of intermolecular hydrogen bonds. Shi et al. obtained a tensile strength improvement of approximately 13% by adding GCN as a reinforcing material into polypropylene [47]. Similarly, Wang et al. added GCN to the epoxy resin as a reinforcement and confirmed that the mechanical properties (tensile strength 73.41 MPa) of GCN were approximately 25–35% higher than those upon the addition of other reinforcements (e.g., graphene, graphene oxide, and surface-modified graphene) at the same content [48].

**Figure 1:**
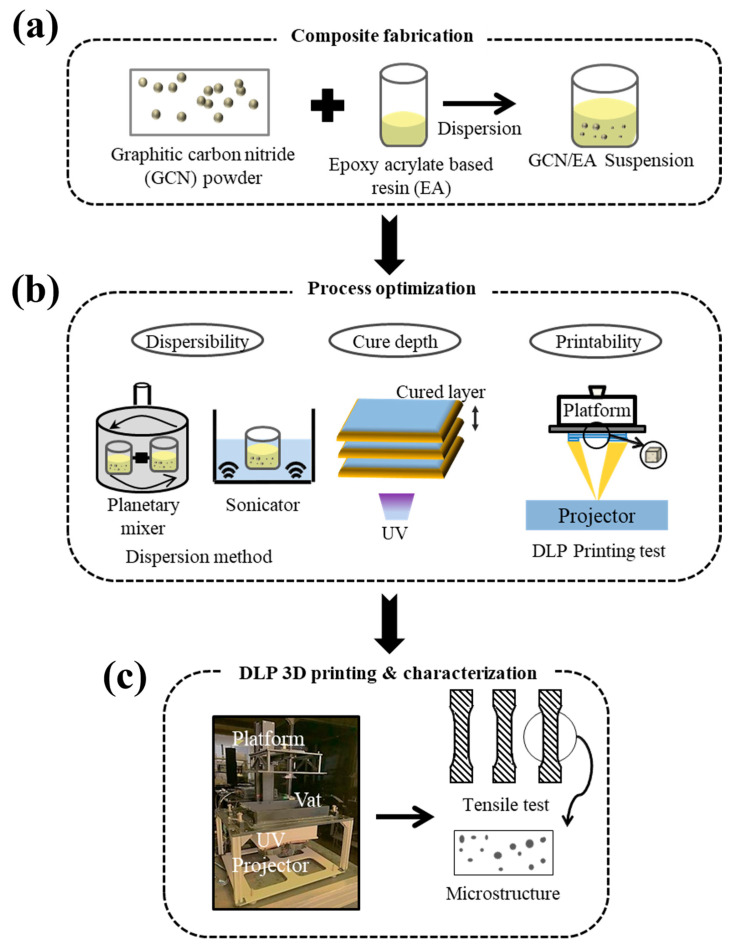
Schematic illustration of (**a**) GCN-EA resin composite fabrication, (**b**) DLP 3D printing process optimization, and (**c**) DLP 3D printing and mechanical strength analysis of the printouts.

In this study, a novel strategy in which GCN could be used as a promising reinforcement material for a 3D-printed polymer matrix to overcome the drawbacks (poor mechanical properties) of the DLP-3D-printed products is introduced. Figure 1 shows the schematic of the entire process. The homogeneous dispersion of GCN into a polymer matrix is considered a key factor [49] because bulk GCN has poor dispersibility in both water and organic media owing to interlayer van der Waals interactions (π–π stacking) that cause agglomeration in the liquid phase [50]. The homogeneous dispersion of GCN in epoxy acrylate (EA)-based polymer resin for DLP 3D printing was achieved by sequential dispersion methods: ultrasonication and planetary mixing; it was confirmed that GCN was homogeneously dispersed in the EA resin. Using the GCN-dispersed EA resin, the cure depth was measured to determine the feasibility of DLP 3D printing and optimize the printing process. The mechanical properties of the DLP-3D-printed GCN/EA composites were analyzed through tensile testing, followed by detailed micro-surface analysis. This study demonstrates a feasible method of adopting an economical and high-performance reinforcement to overcome the lower mechanical strength of DLP-3D-printed products.

## 2. Materials and Methods

### 2.1. Preparation of Materials and 3D Printing Process

A photocurable polymer for DLP 3D printing, bisphenol A-type EA-based polymer resin (H200, Laonix Co., Ltd., Ulsan, Republic of Korea), was used. Bulk GCN powder was synthesized through the thermal condensation of the urea precursor. Herein, 90 g of urea was placed in a 200 mL alumina crucible and heated at 560 °C in a muffle furnace for 2 h, followed by air cooling for 1 h. Bulk GCN powder was milled for 4 h at 200 rpm using a ball mill machine (BML, Daihan Scientific Co., Ltd., Daejeon, Republic of Korea) with mixed zirconia balls (diameters of 1 and 5 mm) for dispersion in the EA resin. Ball-milled GCN powder was added into EA according to different concentrations (cure depth measurement: 0.5–1.5 wt%, tensile specimen: 0.005–0.03 wt%) and dispersed with a planetary mixer (KK-250SE, KURABO Ltd., Tokyo, Japan) at 1000 rpm for revolution and 100 rpm for rotation for 5 min. After planetary mixing, further dispersion was performed for 70 min using an ultrasonicator (Powersonic 400, Hwashin Tech Co., Ltd., Seoul, Republic of Korea).

A DLP 3D printer (EDGE 200, Laonix Co., Ltd.) with a build size of 215 mm × 130 mm × 200 mm, resolution: 2560 mm × 1600 mm, and pixel size: 0.07 mm equipped with an LCD display to build 3D printout. The cure depth of the DLP light source was measured for each GCN content (0.5, 1.0, 1.5 wt%) to confirm the processability and printability of the GCN/EA composite resin. The process parameters of the cure depth were set as follows: coating thickness of 200 μm, light source wavelength of 405 nm, light source intensity of 10 mW/cm^2^, and curing time of 10 s. After the curing depth measurement, a straight-line structure cube frame and a curved gyroid structure at both 1 wt% GCN concentrations were printed using a DLP 3D printer to measure the printability of the GCN/EA composite. In addition, tensile specimens (GCN content: 0.005–0.03 wt%) were subjected to DLP 3D printing to measure and analyze the mechanical properties of the GCN/EA composite. The process parameters of DLP 3D printing were set as follows: light source wavelength of 405 nm, light source intensity of 10 mW/cm^2^, curing time of 10 s for each layer, and layer thickness of 100 μm, which is the same as when the cure depth is measured. All outputs of the DLP 3D printing were post-cured for 4 h using a UV post-curing lamp with a wavelength of 405 nm.

### 2.2. Characterization and Analysis

The structure of the GCN powder was confirmed with X-ray diffraction (XRD, ULTIMA 4, Rigaku, Tokyo, Japan), and the functional groups in the GCN molecules were characterized by Fourier transform infrared spectroscopy (FT-IR, Satellite 5000, Mattson, Fremont, CA, USA). Morphological analysis of the GCN and GCN/EA composites was performed through an optical microscope (OM, RH-2000, Hirox, Tokyo, Japan) and scanning electron microscopy (SEM, S-4700, Hitachi, Tokyo, Japan). Particle size analysis of GCN was carried out using a laser particle size analyzer (LA-960, HORIBA, Kyoto, Japan). The absorbance of GCN at different wavelengths was measured using a UV-vis spectrophotometer (Cary 5000, Agilent, Santa Clara, CA, USA). Tensile tests of the GCN/EA specimens were conducted using a tensile testing machine (Quasar 50, Galdabini, Cardano Al Campo, Italy) with a 10 kN load cell according to ASTM D-638 [51]. The tensile test was conducted at a velocity of 1 mm/min until the specimens broke down. The Young’s modulus was obtained by taking the gradient of the line on two points fitted at 0.0% and 0.2% in the stress-strain plot.

## 3. Results and Discussion

### 3.1. GCN Characterization

**Figure 2:**
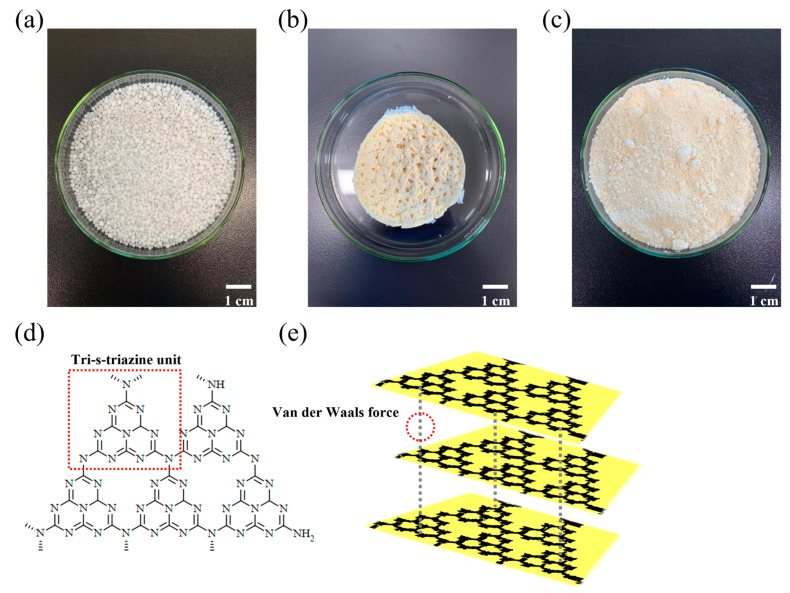
Photographs of different compounds. (**a**) Urea, (**b**) as-synthesized GCN, and (**c**) ball-milled GCN. Molecular structures of GCN in (**d**) 2D and (**e**) 3D. The urea-derived GCN is composed of repetitive tri-s-triazine units and exhibits lamellar structure by interlayer van der Waals interaction.

Yellowish GCN powder was synthesized through the thermal condensation of urea (Figure 2). As shown in Figure 1, GCN is composed of repetitive tri-s-triazine units (Figure 2d) and exhibits a lamellar structure due to weak van der Waals forces (π–π stacking) (Figure 2e) between the GCN layers [52].

The existence of van der Waals interactions in the GCN interlayers inhibits the formation of a homogeneous resin composite for 3D printing, resulting in the agglomeration of GCN in the EA resin. To increase the dispersibility of GCN in the EA resin, physical treatment (such as ball milling, hydrothermal treatment, and ultrasonication) is required to apply physical stress to the agglomerated GCN powder for disassembling it into smaller pieces [53]. Moreover, a chemical modification that functionalizes charged groups on the GCN surface to improve dispersibility by increasing electrostatic is demanded [50]. In this study, a ball-milling process was applied to facilitate the dispersion of GCN powder in the EA resin by increasing the GCN particle surface area and decreasing the GCN particle size. The GCN balling process was observed using SEM, particle size analysis, and FT-IR spectroscopy.

**Figure 3:**
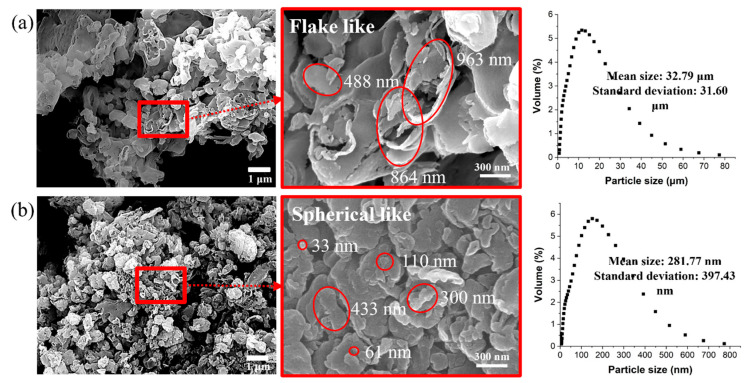
SEM images of (**a**) as-synthesized and (**b**) ball-milled GCN. As-synthesized GCN powders show a flake-like shape with a size of several hundred nanometers. The ball-milled GCN powers have spherical shapes with smaller size diameters.

Figure 3 shows the morphologies of the as-synthesized GCN and GCN ball-milled for 4 h at 200 rpm. Figure 3a shows that the as-synthesized GCN powders have the structure of a bunch of fabric consisting of flake-like shapes of individual GCN particles with a size of hundreds of micrometers. In the case of ball-milled GCN (Figure 3b), it can be confirmed that the GCN powders have a fine spherical-like shape with a minimum particle size of tens of nanometers.

Particle size analysis was conducted to determine the particle size distribution and confirm the mean particle sizes of the as-synthesized and ball-milled GCN powders. The mean particle size of GCN powders dramatically decreased from 32.79 μm (as-synthesized GCN particles) to 281.77 nm (ball-milled GCN particles).

According to previous studies [54,55,56], ball milling is effective in decreasing individual particle size with reduced particle aggregation induced by van der Waals interactions. The drastic decrease in the average ball-milled GCN particle size (less than 1/100) suggests that the van der Waals interaction among the ball-milled GCN particles was reduced.

**Figure 4:**
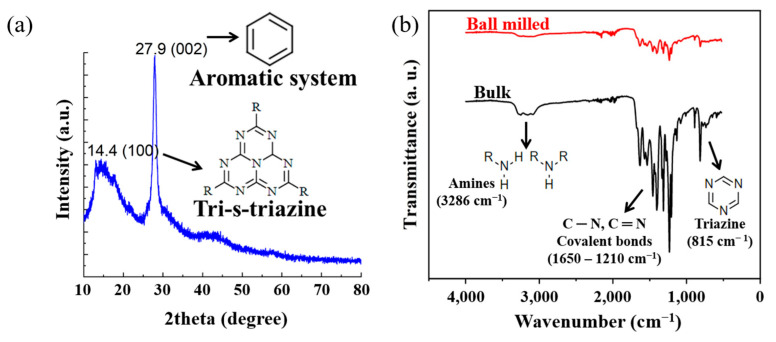
(**a**) X-ray diffraction (XRD) spectrum ball-milled GCN. Graphs show the intrinsic properties of GCN. (**b**) FT-IR spectra of as-synthesized and ball-milled. The type of characteristic peaks was maintained, while the intensity of the peaks after ball milling was weakened.

The XRD results for the structural analysis of ball-milled GCN are presented in Figure 4a; the characteristic peaks of GCN were observed. The peak at 13.1°, which is the value of the interplanar 0.68 nm distance on the (100) plane, appears owing to the regular arrangement of tri-s-triazine units [57]. Another 27.9° peak corresponding to the (002) plane describes the graphitic properties in which the conjugated aromatic molecules are uniformly stacked with an interplanar distance of 0.32 nm [58]. Figure 4b shows the FT-IR analysis, which was obtained for the chemical analysis of as-synthesized and ball-milled GCN. In the FT-IR spectrum, the peak at 3300–3100 cm^−1^ represents a primary or secondary amine group in the heterocyclic molecules [54], and peaks within the range of 1600–1200 cm^−1^ are characteristic of C-N heterocyclic molecules [55]. The vicinity of 800 cm^−1^ indicated the presence of triazine, a unit constituting the tri-s-triazine [56]. On comparing the FT-IR analysis of bulk and ball-milled samples in more detail, there was little dramatic change in the characteristic peaks of GCN powder after the ball-milling process; however, it can be confirmed that the intensity of the characteristic peaks of the C-N covalent bond was weakened by 3% in the ball-milled GCN in relation to that of the as-synthesized GCN (Figure 4b). This result is attributed to the breakage of hydrogen bonds between the triazine rings and amines during the ball-milling process. Therefore, it is expected that the interlayer van der Waals interactions will also decrease because of the breakage of bulk GCN molecules or an increase in the interlayer distance, and accordingly, the particle agglomeration effect of the powder itself can be reduced.

### 3.2. Dispersibility of GCN in EA

**Figure 5:**
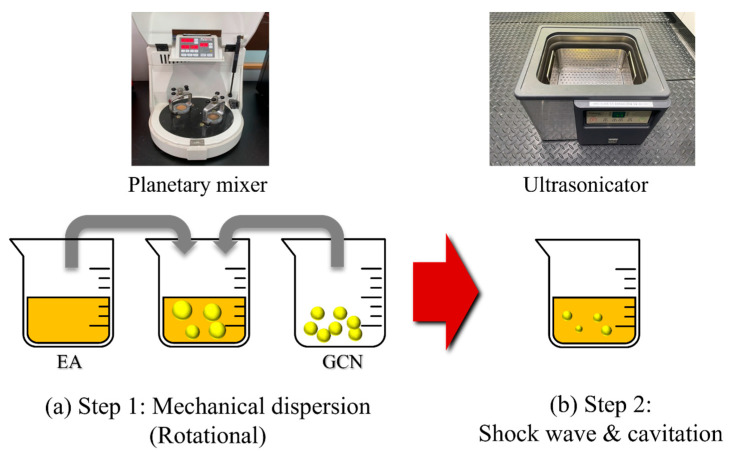
Methods for the dispersion of GCN into EA. (**a**) Step 1: planetary mixing and (**b**) step 2: ultrasonic dispersion. In step 1, mechanical dispersion of GCN in the suspension is performed and in step 2, grinding into finer particles is performed in the suspension.

**Figure 6:**
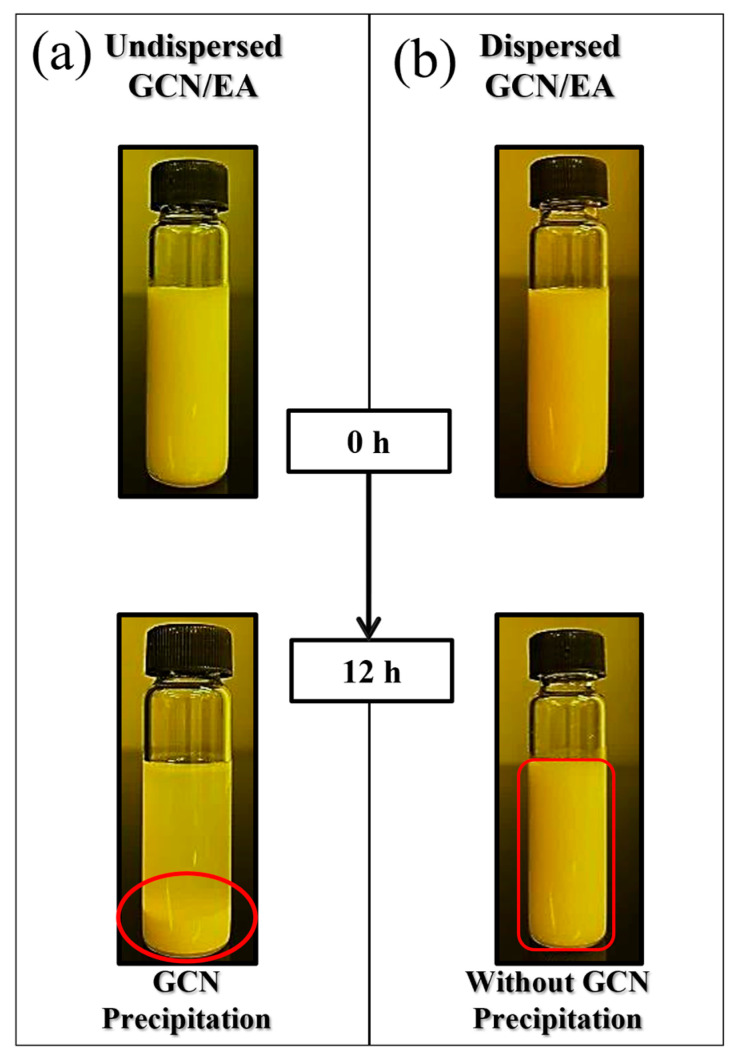
Dispersibility comparison between (**a**) undispersed (red circle, GCN precipitation) and (**b**) dispersed GCN/EA suspension at 1 wt% GCN content (red block, without GCN precipitation).

Two-step dispersion methods were applied to disperse GCN, which is insoluble in EA (Figure 5). In step 1, mechanical dispersion of GCN in the suspension is performed by rotational force. In step 2, grinding into finer particles is performed in the suspension by the shock wave and cavitation effect. Figure 6 shows the dispersibility measurement results of the GCN/EA suspension with 1 wt% GCN content with and without the two-step dispersion method. The undispersed suspension represents a state in which the GCN/EA suspension is simply stirred with a glass rod for 5 min, and the dispersed suspension represents a state in which planetary mixing and ultrasonication dispersion are applied. In the image on the left of Figure 6 of the state immediately after dispersion, no precipitation was observed in either the undispersed or dispersed suspensions. After 12 h, precipitation occurred only in the undispersed suspension, and precipitation hardly occurred in the dispersed suspension. This result suggests that the possibility of agglomeration of the dispersed composite material is low, even during the extended 3D printing process.

**Figure 7:**
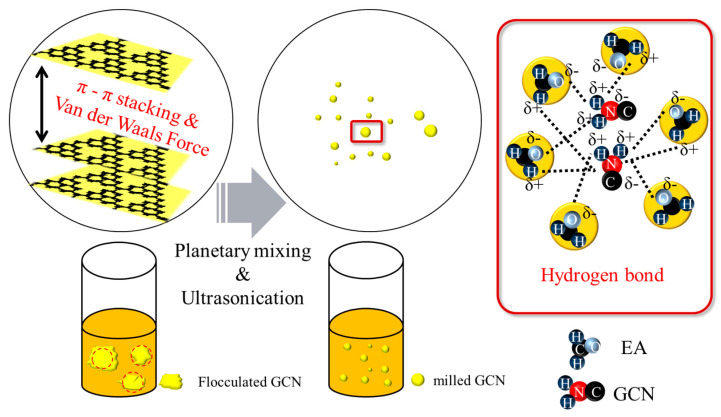
Schematic illustration of dispersion mechanism of GCN in EA. GCN, which was agglomerated by van der Waals force (π-π stacking, red dash circle), is ground by the dispersion process and forms hydrogen bonds with EA (red block). Subsequently, GCN interacts with the EA resin molecules after being separated from each other.

Figure 7 shows the proposed dispersion mechanism of GCN/EA suspensions subjected to planetary mixing and ultrasonication. A large amount of agglomeration would occur because of the interlayer van der Waals attraction and the π–π stacking between the GCN molecules before the dispersion process; however, when the suspension was processed by the planetary mixing and ultrasonication dispersion process, the agglomerated GCN could be uniformly disassembled. This is because the physical energy of two-step dispersion methods destroys the van der Waals interactions, according to You et al. [59]. Thus, it is considered that the uniformly dispersed GCN forms hydrogen bonds and electrostatic attraction with the EA molecules and maintains a uniformly dispersed state for 12 h.

### 3.3. DLP Printability of Dispersed GCN/EA Composite

**Figure 8:**
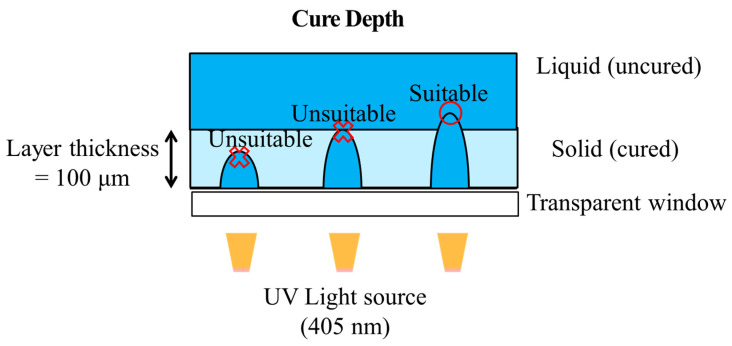
Schematic illustration of cure depth, which is an index for process optimization of DLP 3D printing. Cure depth must be larger than layer thickness.

Prior to DLP 3D printing of the dispersed GCN/EA composite material, the cure depth of the dispersed GCN/EA composite was measured to determine whether the composite could be applied in the DLP 3D printing process (process optimization). The cure depth is an index that indicates the degree to which the light source generated by the DLP UV projector penetrates the photocurable composite material. The curing depth must be larger than the layer thickness of the printing to enable smooth DLP 3D printing (Figure 8). It causes instability in the DLP 3D printing process when the cure depth of the composite is smaller than the layer thickness [60].

**Figure 9:**
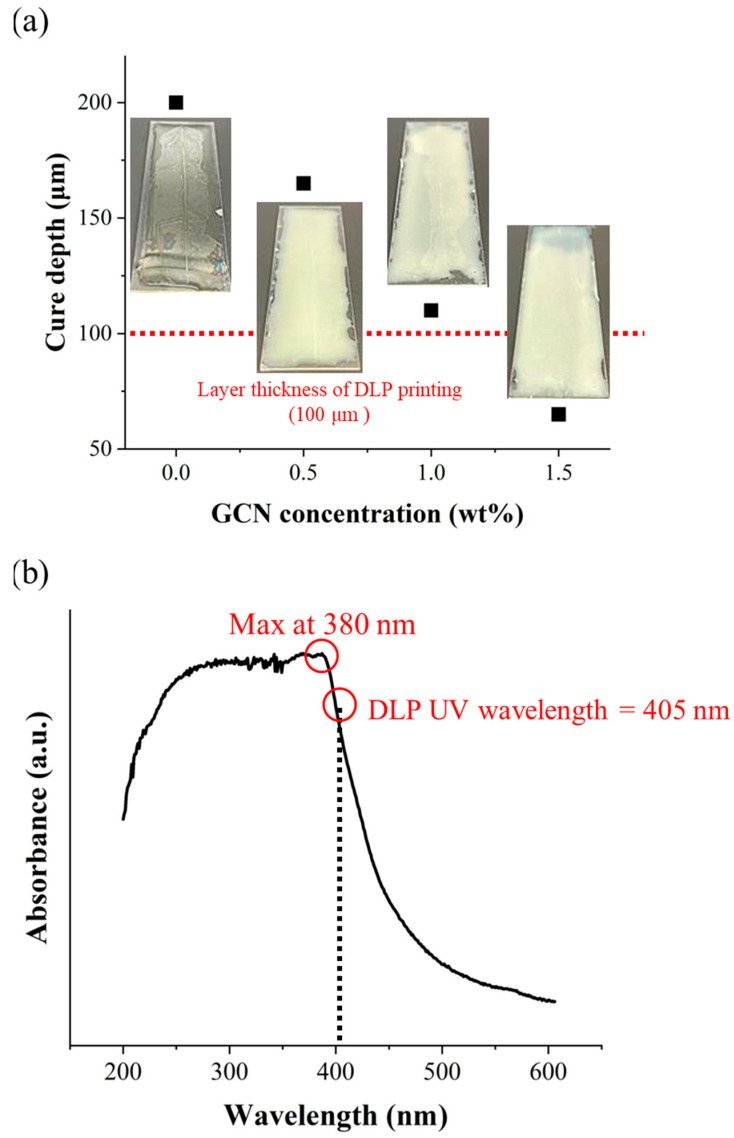
(**a**) Cure depths of dispersed GCN/EA composites with different GCN concentrations. Neat EA: 200 μm, 0.5 wt% GCN/EA: 165 μm, 1 wt% GCN/EA: 110 μm, and 1.5 wt% GCN/EA: 65 μm and (**b**) UV-vis absorption spectrum of GCN. The absorbance of GCN is at its maximum at 380 nm. The absorbance of GCN also exists at 405 nm (the wavelength of the DLP light source), but GCN absorbs a very small amount of UV light.

Cure depth measurements were carried out by coating the dispersed GCN/EA composite according to the different GCN contents with a thickness of 200 μm on a slide glass, irradiating UV with a DLP projector, and measuring the cured thickness. Curing depths of 200 μm for neat EA, 165 μm for 0.5 wt% GCN/EA, 110 μm for 1 wt% GCN/EA, and 65 μm for 1.5 wt% GCN/EA were measured (Figure 9a).

The cure depth decreases as the GCN content increases because GCN can absorb light with wavelengths shorter than 500 nm. The deep penetration and delivery of UV light from the DLP were limited by the absorption of GCN particles in the GCN/EA composite (Figure 9b). Thus, elaborate design, such as the contents of GCN and the degree of dispersion for the GCN/EA composite, can successively implement DLP processes. According to the cure depth results and absorbance spectra for GCN, it is expected that the GCN/EA composite containing less than 1.0 wt% GCN could be applied as a photocurable composite resin for DLP 3D printing because the cure depth value should be larger than 100 μm of the layer thickness.

**Figure 10:**
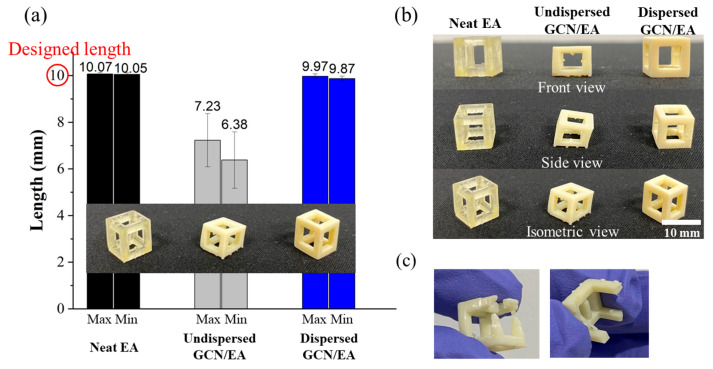
Images of (**a**) Printability measurement (dimensional error), (**b**) appearance of DLP-3D-printed products, and (**c**) exceptional models that did not measure printability.

To confirm the printability of 1 wt% GCN/EA and cure depth of around 100 μm, feedstocks (neat EA, undispersed GCN/EA composite, and dispersed GCN/EA composite) were prepared and printed cube frame structures with a size of 10 mm × 10 mm × 10 mm (individually five samples were prepared). In the DLP 3D printing process, when the material is nonuniformly dispersed, critical shape deformations of the output occur. Because of this deformation, the output is not in the shape of an intended cube shape but in the shape of a slanted cube, which has a long side and a short side at the same time. The lengths of edges for printed cubic structures were measured to verify the dimensional error (Figure 10a). At this time, outputs with cut-off sides or that were not printed to the cube shape were excluded from the measurement (Figure 10b,c). Figure 10a shows that the output of the neat EA and dispersed GCN/EA had a small dimensional error of less than 1.3% from the designed dimension of 10 mm. However, the undispersed GCN/EA outputs had relatively large dimensional errors of 27.7% for the maximum side and 36.2% for the minimum side (Table 1). The standard deviation of the lengths for the five outputs was 0.07 mm on the maximum length, 0.07 mm on the minimum length for the neat EA output, 0.11 mm for the maximum length, and 0.11 mm on the minimum length for the dispersed GCN/EA output. On the contrary, in the case of undispersed GCN/EA output, the standard deviations for the maximum and minimum length sides were 1.14 mm and 1.22 mm, respectively.

Figure 10 and Table 1 show that GCN was well-dispersed within EA through two-step dispersion methods and also demonstrated that the dispersed GCN/EA composite material can be applied in the DLP 3D printing process. In the case of undispersed GCN/EA composite, a large amount of agglomeration occurs owing to inhomogeneous dispersion, and the output is poorly printed because the cure depth is insufficient as agglomerates interfere with the penetration of UV, resulting in dramatic dimensional errors.

**Figure 11:**
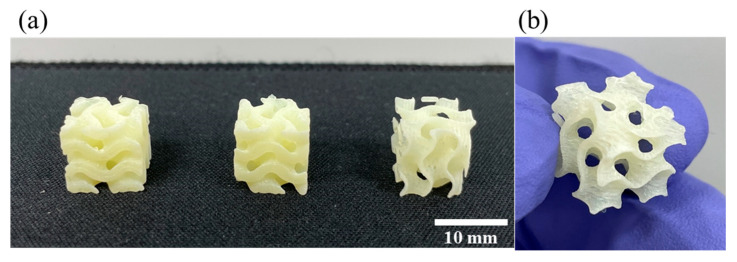
Results of printability measurement of dispersed GCN/EA composite of the curved structure. (**a**) Outputs of gyroid structure and (**b**) magnified view.

In addition to the printability test of the straight-shaped structure, the gyroid structure, one of the constituent elements of triply periodic minimal surfaces (TPMSs) having a large specific surface area [61], was printed using the DLP 3D printing process with GCN/EA composite to demonstrate the printability of the curved structure (Figure 11).

Figure 11 shows that not only linear structures but also curved structures with various line widths (at least 1 mm) are 3D-printed well without deformation with the dispersed GCN/EA composite. The total time required for DLP 3D printing in this experiment was less than 1 h and 30 min, indicating that agglomeration and precipitation of GCN during DLP 3D printing did not occur based on the results of the previously shown dispersibility test in Figure 6.

### 3.4. Mechanical Properties of DLP-3D-Printed GCN/EA Composite

**Figure 12:**
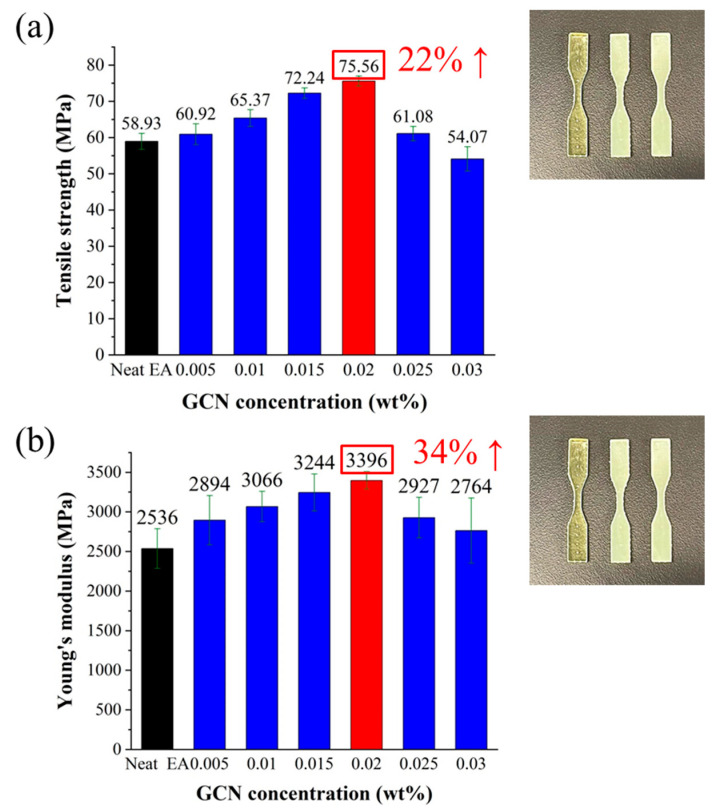
Results of tensile test. (**a**) Tensile strength and (**b**) tensile modulus with different GCN contents.

Tensile specimens of a dispersed GCN/EA composite using a DLP 3D printer were prepared to measure the tensile strength and modulus of elasticity (Figure 12). Both the tensile strength and modulus of elasticity increased as the GCN content increased up to a content of 0.02 wt%. The maximum tensile strength was 75.56 MPa at 0.02 wt% of GCN, which was 22% higher than that of neat EA, and the maximum modulus of elasticity was 3396 MPa, which was 34% higher than that of neat EA.

**Figure 13:**
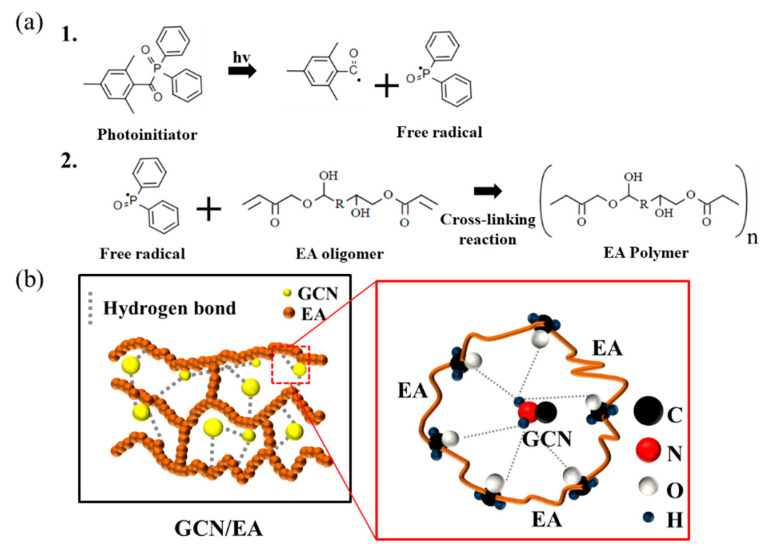
Expected schematic illustration of (**a**) free radical photopolymerization of EA (1. Free radical generation; 2. The radical induced EA polymerization) and (**b**) interactions between EA polymer and GCN molecules. Hydrogen bonds between polymerized EA and GCN molecules reinforce the mechanical properties of EA/GCN composite outputs.

Figure 13 shows the expected mechanism of photopolymerization of the EA resin and the reinforcing effect occurring in the GCN/EA composites. As shown in Figure 13a, the polymerization reaction that occurs during DLP printing of the GCN/EA composite is a radical photopolymerization reaction. In radical photopolymerization, free radicals are created by the photoinitiator, and these free radicals cause the growth of the EA oligomer to the EA polymer.

Simultaneously with radical photopolymerization, fine and uniformly dispersed GCN molecules through planetary mixing and ultrasonication dispersion are interlocked with the polymerized EA polymers to form hydrogen bonds and electrostatic attraction with the EA molecules, thereby increasing the strength of the composite material (Figure 13b).

**Figure 14:**
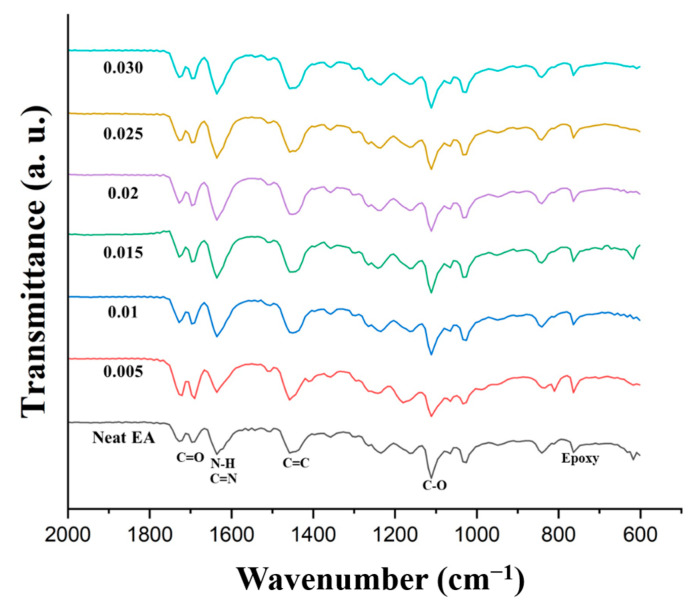
FT-IR spectra of DLP-3D-printed GCN/EA composite and neat EA with different GCN contents.

FT-IR analysis of the DLP-3D-printed GCN/EA composites and neat EA was conducted (Figure 14) to confirm the presence of hydrogen bonds. In the spectrum of neat EA, several characteristic peaks were observed: epoxy groups (833 cm^−1^), C-O groups (1110 cm^−1^), C=C groups (1458 cm^−1^), N-H and C=N groups (1635 cm^−1^), C=O groups (1689 cm^−1^), and aliphatic C-H groups (2924 cm^−1^) [62,63,64]. FT-IR result indicates that as the GCN content increases, C=N and N-H peaks of bending vibration increase. This is evidence of the mechanism of hydrogen bond formation between GCN and EA (Figure 13). However, as the GCN content increased, the degree of hydrogen bonding increased; however, as the number of GCN particles increased, the crosslinking density decreased. Therefore, to obtain optimal mechanical strength, it is necessary to determine the appropriate GCN concentration for the reinforcement.

**Figure 15:**
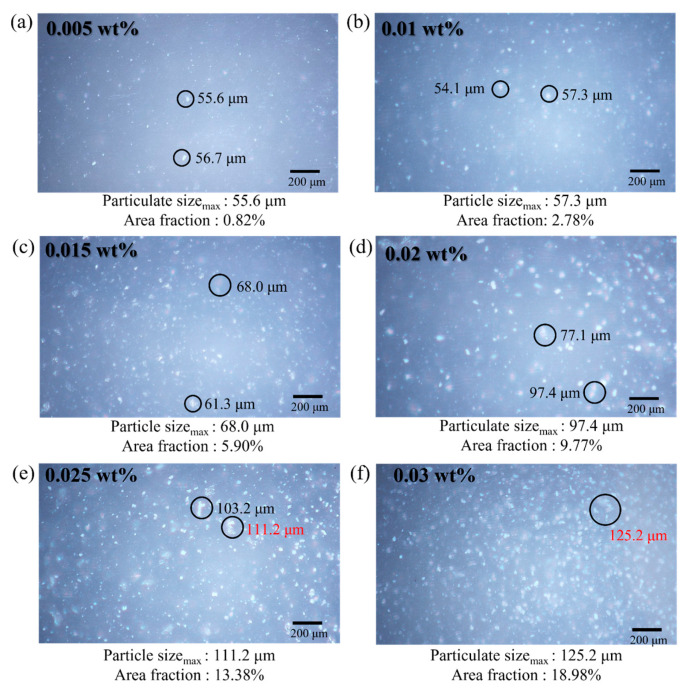
Surface micrographs of DLP-3D-printed specimens (EA/GCN composites) for each GCN loading. (**a**) 0.005 wt%, (**b**) 0.01 wt%, (**c**) 0.015 wt%, (**d**) 0.02 wt%, (**e**) 0.025 wt%, and (**f**) 0.03 wt%.

As shown in Figure 15, the number of GCN particles increases with the increment of GCN contents, leading to a higher possibility of crack deviation. However, it is observed that the GCN contents of more than 0.025 wt% have exceeded the appropriate amount to inhibit crack propagation owing to excessively formed GCN agglomerates in the EA matrix. Therefore, the affinity between GCN particles and EA molecules is decreased, thereby reducing the crack deviation effect.

To observe the distribution of GCN with different GCN contents in the GCN/EA tensile specimen, the size and area fraction of the GCN particulates were observed with OM (Figure 15). The maximum particulate size and area percentage for each GCN content were 0.005 wt%:56.7 μm, 0.82%; 0.01 wt%:57.3 μm, 2.78%; 0.015 wt%:68.0 μm, 5.90%; 0.02 wt%:97.4 μm, 9.77%; 0.025 wt%:111.2 μm, 13.38%; 0.03 wt%:125.2 μm, 18.98% and it was confirmed that GCN was uniformly distributed in all-optical microscopic images. Within 0.005–0.02 wt% GCN contents, particulates of less than 100 μm were observed, and the effect of the strength reduction by the agglomeration of GCN particles is insignificant in relation to the degree of strength improvement due to the hydrogen bond interaction formed between GCN particles and EA molecules. However, at a GCN content of over 0.02 wt%, the size of the GCN particulates gradually increased to more than 100 μm. Particularly, at 0.03 wt%, particulates of up to 125 µm and a GCN area percentage of 18.98% were observed. The tensile strength at 0.03 wt% was 54.07 MPa, which was significantly lower than the maximum tensile strength of 75.56 MPa (GCN content of 0.02 wt%).

## 4. Conclusions

GCN has been utilized as a novel reinforcing material for improving the mechanical properties of photocurable EA polymers for DLP 3D printing. Planetary mixing and ultrasonication dispersion were applied to disperse insoluble GCN in the EA resin. Dispersibility observation confirmed that the degree of sedimentation of the dispersion-treated suspension over time was improved. In addition, printability was examined by cure-depth analysis and tested by applying the dispersed GCN/EA composite to DLP 3D printing. The printing output of the dispersed GCN/EA composite showed a small dimensional error of 0.3–1.3%, confirming that it was robustly printed as designed. In addition, the tensile strength and elastic modulus of 3D-printed GCN/EA composites (0.02 wt% of GCN) were 75.56 MPa and 3396 MPa, respectively, which are 22% (tensile strength) and 34% (elastic modulus) higher than those of neat EA specimens. By observing the failure cross-section of the tensile specimens, it can be concluded that the reinforcement (GCN) distribution (i.e., particulate size and portion) in a matrix (EA) would determine the mechanical properties of composites (GCN/EA). In addition, as a follow-up study, there are attempts to apply surface-modified GCN as a functional material for DLP 3D printing to evaluate the applicability of the DLP 3D printed GCN/EA composite in optics, displays, and photocatalysts.

This approach is capable of DLP 3D printing to emerge as a greatly practical manufacturing process. The proposed approach suggests that economic reinforcements can be applied to widely used 3D printing materials to develop functional 3D printing feedstocks. Moreover, with higher mechanical properties, it could expect to meet the requirements of the industrial demands for 3D printed plastic products. Consequently, the DLP printed composites are expected to be used more in a variety of industrial fields such as automobile, shipbuilding, and aviation industries.

## Figures and Tables

**Table 1:** Results of dimensional error in printability test. Max means maximum side length, and Min means minimum side length.

	Neat EA		Undispersed		Dispersed	
	Max	Min	Max	Min	Max	Min
Difference from designed dimension (%)	0.7	0.5	27.7	36.2	0.3	1.3

## Data Availability

Data are contained within the article.
